# Partial Resection of the Lung

**Published:** 1919-01

**Authors:** 


***Partial Resection of the Lung.*** By M. Gregoire. *La Presse Médicale,* October 3, 1918.

A soldier wounded in the lung presented some signs of pleural and pulmonary infection. A thoracotomy having been practised for the removal of the projectile, a tumor was discovered on the inferior margin of the right lung.

The tumor was as large as a small egg, of a regular shape, and dark red; a bullet could be felt in its center. By puncturing, it was determined that this tumor was an abscess developed around the missile.

Instead of draining out the pus, fearing that this might be accompanied by the infection of the pleural cavity, M. Gregoire decided to remove altogether the abcess and the surrounding part of the lung. This having been pulled out by means of heart shaped forceps, and the pleura being carefully protected by "champs", he removed the whole region around the abscess, cutting off the lung's parenchyma along a curved line, and taking off about 10 cm. of the inferior margin of the lobe to a depth of 6 or 7 cm.

Moderate hemorrhage.

Subsequent suturing, with catgut, of the resected section and the wall.

On the following day, a subsequent pneumothorax was punctured. The patient recovered quite normally, and left the hospital one month later.

